# Primary biliary cirrhosis with refractory hypokalemia: A case report

**DOI:** 10.1097/MD.0000000000013172

**Published:** 2018-11-30

**Authors:** Kai-Hui Dong, Yi-Na Fang, Xiao-Yu Wen, Qing-Long Jin

**Affiliations:** Department of Hepatology, The First Hospital of Jilin University, Changchun, China.

**Keywords:** hypokalemia, primary biliary cirrhosis, renal tubular acidosis

## Abstract

**Rationale:**

Renal tubular acidosis (RTA) represents a class of metabolic disorders characterized by metabolic acidosis with a normal plasma anion gap. As a rare complication of primary biliary cirrhosis (PBC), RTA is easily overlooked, likely leading to misdiagnosis.

**Patient concerns:**

A 32-year-old woman who had been diagnosed with PBC at our hospital was found to have hypokalemia due to repeated fatigue for 2 years, and the etiology was unknown.

**Diagnoses:**

Due to the laboratory test results, radiographic findings, and pathologic results, she was diagnosed with PBC associated with RTA.

**Interventions:**

She was then treated with ursodeoxycholic acid, potassium citrate, and calcium supplements together with activated vitamin D.

**Outcomes:**

Thus far, the patient showed a good response to ursodeoxycholic acid, and the clinical symptoms and liver function were significantly improved.

**Lessons:**

Physicians that encounter refractory hypokalemia in a patient with PBC should be aware of the presence of RTA. The early diagnosis and treatment of such patients are of paramount importance to alleviate clinical symptoms and delay disease progression.

## 1 Introduction

Primary biliary cirrhosis (PBC) is an autoimmune-mediated, chronic, progressive cholestatic liver disease of unknown etiology.^[1]^ Renal tubular acidosis (RTA) represents a class of metabolic disorders caused by various types of renal acidosis dysfunction.^[2]^ Severe hypokalemia is a central feature of the classic distal RTA, both in hereditary and acquired forms.^[3]^ However, RTA is rarely documented as a complication of PBC, and is easily misdiagnosed due to ignorance of the etiology of hypokalemia. Here, we report an interesting case of PBC with refractory hypokalemia to raise awareness of PBC with RTA. The patient provided informed consent for the data concerning the case to be submitted for publication. This case report was approved by the ethics committee of our hospital.

## 2 Case report

**Figure 1:**
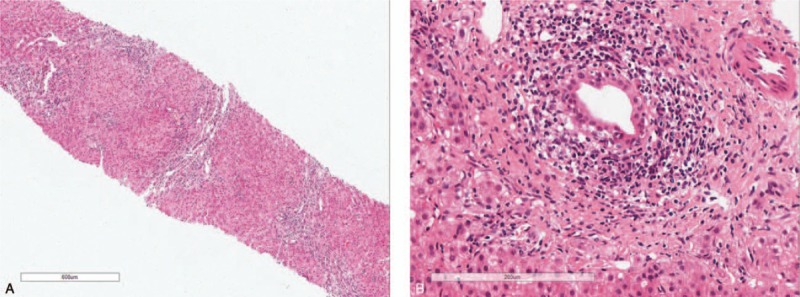
(A and B) Liver biopsy results show moderate interface hepatitis and nonsuppurative destructive cholangitis.

In spring of 2013, a 32-year-old woman sought medical attention at the Jilin University First Bethune Hospital with complaints of intermittent pruritus and fatigue. Liver function tests revealed significantly increased levels of γ-glutamyltransferase (γ-GT, 468 U/L) and alkaline phosphatase (ALP, 968 U/L). The patient was diagnosed with hypothyroidism 3 years ago due to the use of ^I31^I therapy and was treated with euthyrox (250 mg/d). She had no hypertension, diabetes, special drug, hepatitis, or consumption of alcohol history, as well as no any family history. No positive signs were observed on physical examination. Serology for hepatitis virus A, B, or C was negative. Immunologic tests showed that serum antinuclear antibodies were positive (1:640), accompanied by increased concentrations of serum immunoglobulin M (IgM, 5.01 g/L) and the presence of an anti-mitochondrial M2 antibody (AMA-M2, >200 RU/mL); however, the anti-Ro (SS-A) and anti-La (SS-B) antibodies were negative. A liver biopsy showed stage II PBC (Fig. 1A and B). The patient was eventually diagnosed with PBC and was treated with ursodeoxycholic acid (UDCA, 250 mg/d). The cholestatic enzyme levels and symptoms of pruritus were significantly improved.

In April 2016, the patient visited our hospital because of repeated fatigue for 2 years. Liver and kidney function, serum glucose levels, and blood routine tests were normal. Laboratory tests mainly revealed that the level of serum potassium (2.42 mmol/L) was low. The patient was treated intermittently with potassium citrate. However, the therapeutic effect was not satisfactory, and the cause of low potassium was unknown.

In July 2016, the patient visited the superior hospital for further treatment. She weighed 43 kg, and her height was 155 cm. Blood tests showed the following: pH, 7.347; PaCO_2_, 33.1 mm Hg; HCO_3_^−^, 17.7 mmol/L; PaO_2_, 103 mm Hg; Na^+^, 141 mmol/L; K^+^, 3.4 mmol/L; Cl^−^, 113 mmol/L; Mg^2+^, 0.94 mmol/L; serum creatinine, 70 μmol/L. In addition, the plasma anion gap (AG) was normal: AG = [Na^+^] – [Cl^−^ + HCO_3_^−^] = 141 – (113 + 17.7) = 10.3 mmol/L (normal, 10–14 mmol/L). Urine routine showed a pH of 7.5, and 24-hour urine analysis demonstrated that the urine potassium concentration was 94 mmol/24 h. The aldosterone renin ratio was normal. Baseline data of the patient are summarized in Table 1. An abdominal ultrasound showed evidence of cirrhosis and gallstones. Adrenal CT showed bilateral nephrocalcinosis and no tumor. Whole-body bone mineral density was 0.99 g/cm^2^ with a T score of −1.3. According to the patient's history and related test results, she was finally diagnosed with RTA in PBC and was then treated with a combination of UDCA, potassium citrate, calcium supplements and activated vitamin D. One year after the treatment, the patient's liver function remained stable, and the clinical symptoms were significantly improved.

**Table 1:**
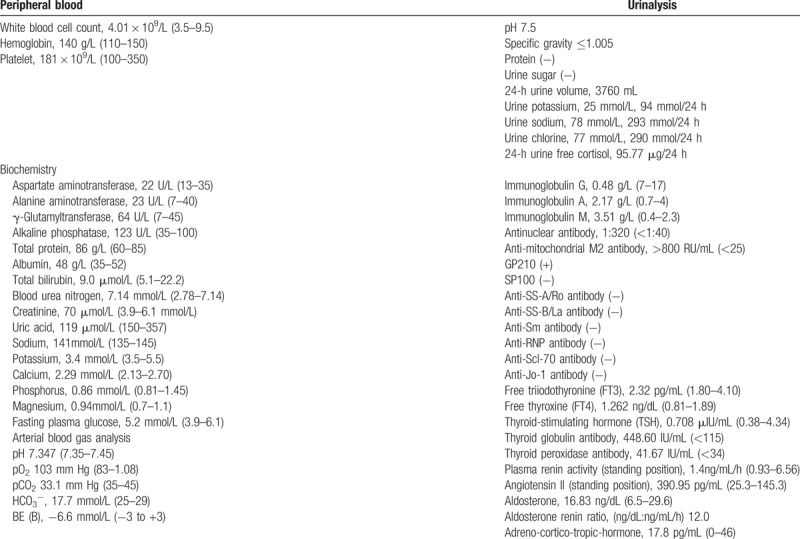
Baseline data of the patient in July 2016.

## 3 Discussion

The PBC is an immune-mediated, chronic, cholestatic liver disease that predominantly affects middle-aged women, with a male to female ratio of 1:10, and is characterized by nonsuppurative destruction of small intrahepatic bile ducts, leading to fibrosis and potential cirrhosis as complications.^[4]^ The PBC incidence rates range from 0.33 to 5.8 per 100,000 inhabitants/year, and the prevalence rates range from 1.91 to 40.2 per 100,000 inhabitants and increase over time.^[5]^ The pathogenesis of PBC has not yet been fully elucidated. The most common symptoms of PBC are fatigue and pruritus. Chronic cholestasis can lead to steatorrhea and lack of various fat-soluble vitamins, resulting in conditions such as osteomalacia and osteoporosis. The PBC diagnosis^[6]^ was defined based on the presence of at least 2 of the following criteria: biochemical evidence of cholestasis: mainly elevated ALP; positivity for AMA; liver histologic examination for nonsuppurative destructive cholangitis and interlobular bile duct destruction.

It has been reported that 70% of PBC patients may have extrahepatic-related diseases, and RTAs are rare but important complications of PBC.^[7]^ RTA, primarily genetic defect diseases, is characterized by the kidney's inability to secrete hydrogen ions or reabsorb bicarbonate ions, resulting in a state of normal AG hyperchloremic metabolic acidosis.^[8]^ The common clinical manifestations of RTA are as follows: hyperchloremia, normal anion interstitial RTA, electrolyte imbalance, bone disease, and urinary tract symptoms. RTA is classified as a group of four distinctive types. Type I RTA, as the most common type in China, is also known as distal RTA, characterized by the distal defect of hydrogen ion secretion, resulting in a urinary pH > 5.5.^[9]^ Long-term acidosis not only leads to osteolysis but also inhibits the reabsorption of calcium ions by renal tubules, leading to osteoporosis. In addition, free calcium can cause nephrocalcinosis or nephrolithiasis, which is related to the kidney's inability to acidify urine. The clinical manifestations of type I RTA are usually related to the disease type and severity and whether it is acquired or inherited.^[10]^ The major diagnostic criteria of type I RTA^[8,11]^ are as follows: normal AG hyperchloremic metabolic acidosis; hypokalemia; and alkaline urine (pH > 5.5). If hypocalcemia, nephrocalcinosis, rickets, or osteomalacia occur, the diagnosis of type I RTA is more supported. In addition to the typical clinical manifestations of RTA described above, patients can also present without symptoms. Ammonium chloride loading tests are required for atypical cases or incomplete distal RTA. Type II RTA occurs due to the inability of the proximal tubule to reabsorb bicarbonate ions. Compared with distal RTA, the main differences between the clinical manifestations of type II RTA include that the bicarbonate ions are increased in urine, the urine pH is below 5.5, and the incidence of urinary stones and renal calcification is lighter than that of distal RTA. Other varieties of the disease include type III mixed, and type IV is secondary to aldosterone deficiency or resistance and is the only type known to be associated with hyperkalemia.

The patient in our case met the above diagnostic criteria for PBC and type I RTA. Therefore, the cause of hypokalemia in this patient was type I RTA, and the presence of type I RTA was secondary to PBC.

Treatment of type I RTA includes the correction of hypokalemia and acidosis, as well as prevention of kidney stones and bone disease^[12]^; secondary distal RTA with a clear cause should seek to remove the cause. Hypokalemia should be corrected first because alkali replacement can worsen hypokalemia with dangerous consequences.^[11]^ Potassium citrate is recommended because kalium chloratum can increase the risk of hyperchloremic acidosis. Correcting hypokalemia improves musculoskeletal symptoms if present.^[13]^ Early treatment also reduces the incidence of nephrocalcinosis, recurrence of renal stones and progression to chronic kidney disease.^[14,15]^ Yamaguchi et al^[16]^ reported that steroid therapy is beneficial to treat renal complications in patients with PBC. Oguejiofor et al^[11]^ found that amiloride, as a potassium-sparing diuretic, can be used to treat severe refractory symptomatic hypokalemia associated with type I RTA. However, the above 2 treatment methods require considerable experimental data for verification. Therefore, we need to further explore the pathogenesis and treatment of RTA with PBC.

In summary, RTA demonstrates complex clinical manifestations and is often misdiagnosed. Therefore, when patients with PBC have uncorrectable hypokalemia, we should focus on the cause of the disease and consider RTA associated with PBC. An early diagnosis and early treatment can delay disease progression and improve the prognosis.

## Author contributions

**Data curation:** Kai-Hui Dong.

**Formal analysis:** Kai-Hui Dong, Yi-Na Fang.

**Resources:** Xiao-Yu Wen, Qing-Long Jin.

**Writing – original draft:** Kai-Hui Dong.

**Writing – review & editing:** Xiao-Yu Wen, Qing-Long Jin.
